# The Immediate Treatment of Accidents and Sudden Illness

**Published:** 1888-09-01

**Authors:** William Moore

**Affiliations:** K.C.I.E., late Surgeon-General with the Government of Bombay.


### The Immediate Treatment of Accidents and Sudden Illness.

By Sir William Moore, K.C.I.E., late Surgeon-General with the Government of Bombay.

Although there are various books detailing the course which should be pursued when persons meet with accidents, or are seized by sudden illness, it does not appear that the information so conveyed reaches the general public, or, as some orators put it, "the masses." Perhaps the information thus given is couched in too technical language. Perhaps the books referred to, being published by medical agents, are overlooked by "the masses." Perhaps, although many people are fond of reading medical works (and often perplex themselves by so doing), the majority confine themselves to studying books treating of the particular ailment they suffer from, or fancy they suffer from. Be this, however, as it may, the fact remains that lamentable public ignorance is patent as to the means which should be adopted in cases of sudden accident or llness. Yet it is most desirable that all persons—and policemen especially—should be acquainted, to some extent at least, with the immediate treatment of accident and sudden seizures. If this were the case, it is not too much to say that many maladies and injuries would be prevented from becoming worse, and that lives would be certainly saved. The Hospital being a journal reaching all classes, appears a suitable medium for the promulgation of elementary information on these important points. "Life is a great gift," and there is no manner of doubt that life might often be preserved were the proper means adopted when it is placed in jeopardy. Yet not one in one hundred apparently knows what to do. Only the other day the writer was passing Kensington Gardens, when he observed an old man being supported by two other persons, and encouraged to cling to the railings, so as to maintain an upright posture. A glance showed what was the matter with the old man. He had been struck by cardiac failure, or serous apoplexy, or that variety of the malady in which the face is pale, and the circulation of the blood failing. His pulse was feeble and intermittent, and there was evident paralysis on one side of the body. Now what ought to have been done in such a case? The poor old man should have been laid on his right side full length, his collar should have been opened, he should have been allowed plenty of fresh air, and his legs, feet, and hands should have been well rubbed. Then when the first shock of the malady had passed away, he ought to have been carried, still in the recumbent posture, to the nearest hospital. Again another instance. Only a few days back the writer was passing down the Hammersmith Road, when two boys were knocked down by a cab. One escaped with little injury, the other got concussion of the brain. He was almost senseless, speechless, cold, and pallid. The same plan ought to have been pursued as in the case of the old man; but instead of this the boy was surrounded by a dense crowd of inquisitive people, and one good but mistaken Samaritan was supporting the injured youth in an upright position on his knee. After a time the poor boy was dragged away on his legs, being well shaken and jostled in the process. Yet a third instance. In Hyde Park, one recent Sunday, a person was taken in an epileptic fit. One bystander wisely ran for water; others carried the epileptic to a bench, on which he was seated. Now, in epilepsy there is great struggling, the limbs are convulsed, and the tongue is very liable to be protruded between the teeth and severely bitten. This accident should be guarded against by placing something, as a small piece of soft wood, too large to slip into the mouth, between the teeth; and the person should be simply prevented injuring himself in his struggles until the fit passes away. But nothing of the kind was attempted; the consequence being that the tongue was bitten, and the limbs of the epileptic were bruised against the hard wood of the bench on which he was seated and held. Had he been placed on the soft grass and left alone, he would have injured himself less.

The following are rules which may be safely adopted in cases of accident and sudden illness. Place the person on the ground or floor lying towards the right side, and with the head raised to the level of the body by a pillow, folded coat, or other soft substance. Then unbutton or split open any clothing pressing upon the neck or chest. The face and chest should be sprinkled with cold water, and some cold water may be given to drink if the power of swallowing remains. Wine, brandy, or other liquor should not be hastily given without evidence of their being needed. Examine the head and limbs separately. The prominent parts of the limbs may be examined with very little movement of the body. If it is necessary to move a person after any injury, especially of the head, the person should be carried while lying down. He should not be allowed to sit upright in a vehicle, or to walk. See that the person is allowed plenty of fresh air. The history of the accident should be obtained, and when sending for a surgeon the message should be as clear as possible, and, if practicable, a written one.

The most common maladies which occur suddenly to people in the streets of large towns are apoplectic and epileptic attacks, maladies depending on heart affections, and drunken fits; although perhaps the latter should not be classed under the head maladies. But it is necessary to distinguish apoplexy and epilepsy from "dead drunk," as it is called, and also from poisoning by opium. Apoplexy is known from epilepsy by the puffing or snoring breathing which occurs in the former malady. In epilepsy there is also struggling of the limbs, the eyes are turned up under the lids, so that the whites only are visible, and the person generally falls down with a loud cry, none of which are the symptoms of apoplexy. Apoplexy is to be distinguished from affections due to the heart, because in the latter maladies the symptoms are very similar to those of fainting. The person is pale, unconscious, with feeble pulse, scarcely perceptible breathing, white lips, and a death-like expression of countenance; while in apoplexy, although the person is unconscious, there is snoring or puffing breathing, and the face is often drawn to one side. Apoplexy is best distinguished from the effects of spirituous liquors by the history of the case, which may probably be one of drinking. Secondly, by the smell of liquor in the person's breath; although this is not a certain sign, for some one may in mistaken kindness have given the person struck with apoplexy some kind of liquor. Thirdly, in the "drunken fit" the size of the pupils of the eyes is equal, while in apoplexy one is often larger than the other. By the pupils of the eyes is meant the round space in the centre of the dark part of the eye. Fourthly, the person "dead drunk" may generally be roused when he babbles incoherently, while from apoplexy the person cannot be roused. Lastly, if any movements occur in drunkenness they will be of all the limbs, whereas movement is usually restricted to one side of the body in apoplexy. From poisoning by opium apoplexy is best distinguished by the history of the case; by the smell of opium in the breath; by both pupils of the eyes being very small and contracted; by the fact that the patient may be roused, although he does not then babble as in the drunken fit, but immediately goes to sleep again; by the fact that apoplexy is most common in advanced life, while opium poisoning is most usual in the young.

It has been stated that "a little knowledge is a dangerous thing," and we are advised not to drink of it unless we imbibe deeply. But there is no rule without an exception, and if we cannot all be what Bacon called "a full man," there is a certain amount of elementary instruction on various matters which it is advisable all should receive. Although those who aim at being their own doctors generally come to grief, there are times when the doctor is not at hand, and when the knowledge of what not to do may be the salvation of the patient. If anyone reading this article remembers what has been said when the time for action or inaction arrives, the little knowledge he possesses will prove the reverse of "a dangerous thing." There is more harm done to injured and sick people by officious mistaken kindness than by actual neglect. Although we are as a nation "brusque," and inclined even to take our pleasures sadly, there is probably more real kindness of heart in the British public than in the public of other ostensibly more polite nations. This is exemplified in the street casualties of London, and especially among the poorer classes. All are anxious to assist, and all would assist if they knew what to do. Many do assist although not knowing the proper course of procedure. If all that has been advanced cannot be held in recollection the condensed rules may certainly be remembered, viz., first, in all cases of accident or sudden illness let the person lie down, and do not assist him to maintain the upright posture; secondly, let him have plenty of fresh air; thirdly, unloose all clothing round the neck and chest; fourthly, convey the patient, still in the recumbent position, to the nearest hospital. Stretchers for this purpose ought to be available at known points, or at least at every police station.

